# A Cross-Sectional Study Assessing Saudi Population's Awareness Toward the Relationship Between Oral Health and Overall Well-Being

**DOI:** 10.7759/cureus.73880

**Published:** 2024-11-17

**Authors:** Ibraheem K Bamaga, Aseel Almalki, Jomanh Alnafei, Nouf Alsubhi, Shahinaz Sembawa, Amani M Harrandah

**Affiliations:** 1 Basic and Clinical Oral Sciences, Umm Al-Qura University, Makkah, SAU; 2 Preventive Dentistry, Umm Al-Qura University, Makkah, SAU

**Keywords:** awarness, knowledge, oral health, overall well-being, public health

## Abstract

Background and aim: Oral health constitutes a critical component of overall health. Additionally, there is evidence of a bidirectional interaction between oral health and the progression of chronic diseases. Many chronic illnesses present oral manifestations and thus can impact oral health but conversely, oral health, particularly the presence of oral bacteria, can influence the progression of chronic diseases. Consequently, a comprehensive understanding of this relationship is essential, as it has significant implications for both oral and overall health. This study aimed to evaluate the knowledge of the Saudi population about oral health and its association with systemic diseases, as well as the factors that might affect this knowledge including demographics and educational level.

Methods: A structured questionnaire using multiple-choice questions was developed in Arabic language and distributed among patients visiting the Dental Teaching Hospital of Umm AlQura University. An electronic version of the questionnaire was developed using Google Forms for distribution through social media outlets. The data were analyzed using SPSS, version 29 (Armonk, NY: IBM Corp.). Descriptive statistics, chi-square test, and Fisher’s exact test were used to analyze the data, and the significance level was set at p<0.05. Participants were considered to have good oral health knowledge and awareness if they answered 60% of the questions correctly.

Result: Most participants were female (66.5%), predominantly aged 20-29 years. More than half of the participants (166; 52%) reported insufficient knowledge regarding oral health. Additionally, there was a significant association between gender and the level of knowledge (p<0.001), with females exhibiting a higher level of knowledge compared to males. Furthermore, there was a significant association between the source of information and the level of knowledge (p=0.009) with participants who reported social media as their primary source of information being more likely to have sufficient knowledge.

Conclusion: Since more than half of the surveyed individuals demonstrated a deficiency in oral health knowledge, there is a necessity for enhanced awareness and educational initiatives regarding the implications of oral health on general health within the Saudi population.

## Introduction

Oral health is a crucial element of overall health. The association between oral health and overall health is complex, as chronic oral diseases might negatively affect overall health, and poor oral health is common among medically compromised patients. Poor oral health is linked to various chronic diseases including chronic liver conditions, particularly in non-alcoholic fatty liver cases [1,2]. Individuals with liver disease often display subpar oral health, necessitating careful dental care planning. The progression of liver disease may worsen oral health, with inadequate pre-liver transplant oral health correlates to a higher risk of post-transplant acute rejection. Additionally, poor oral health is associated with elevated ALT levels and lower albumin levels, impacting post-transplant liver health. Consequently, early attention to oral and dental issues in the course of liver disease is crucial, emphasizing the importance of maintaining optimal oral hygiene [3,4].

Another example is that asthmatic patients are known to have poor oral health due to the constant use of steroidal inhalers [5]. The inhaler affects salivary flow rates causing dryness in the oral cavity. Poor oral health is linked to systemic inflammatory disorders like asthma, increasing cytokines such as IL-1β, IL-6, and TNF-α. Asthmatic patients may experience gingivitis due to an altered immune response and mouth breathing-induced dehydration [6]. Furthermore, it has been reported that inhaled or topically applied corticosteroids by asthmatic patients can cause oral candidacies [7].

Furthermore, diabetes is associated with multiple oral diseases including periodontal disease, tooth loss, xerostomia, caries, burning mouth disorder, taste, salivary gland dysfunction, and delayed wound healing [8]. Oral health professionals address issues like periodontitis that affect glycemic control in diabetic patients [9]. Interestingly, it appears that medically compromised patients lack the required knowledge to maintain good oral health. For example, it has been reported that parents of diabetic children have less knowledge regarding diabetes and oral health compared to parents of healthy children. Moreover, diabetic patients have insufficient oral health knowledge, poor oral health attitudes, and fewer dental visits [10,11].

A recent study revealed the significant prevalence of gingival diseases and dental caries in pregnant women, with an average of seven decayed teeth serving as a potential precursor to dental issues in newborns through bacterial transmission [12]. Another study proposed the effective enhancement of oral health status and behaviors in pregnant and lactating mothers through educational interventions during and after pregnancy delivered by oral health professionals [13]. However, it appears that many pregnant women lack knowledge regarding the association between oral health and pregnancy [14,15].

Poor oral health, particularly among patients with chronic kidney disease (CKD), is associated with systemic complications. CKD lacks distinctive oral manifestations but periodontitis in CKD patients is linked to heightened morbidity and mortality due to various related complications [16].

Individuals with cardiovascular disease (CVD) often report oral health issues, unaware of the periodontal disease and CVD correlation [17]. Lack of knowledge extends to potential oral side effects from cardiac medications, like hyperplasia or gingival expansion with calcium channel blockers, increasing the risk of tooth loss due to heightened periodontal disease risk [18].

As mentioned above it appears that although oral health is a crucial part of overall health specifically among medically comprised patients, the knowledge regarding this connection does not seem to be sufficient even among these patients. To our knowledge, no previous study has evaluated the knowledge regarding connection between multiple chronic diseases including diabetes, cardiovascular diseases, asthma, liver diseases and pregnancy, and oral health among the Saudi population.

Therefore, this study aimed to evaluate the knowledge and awareness of the general Saudi population towards oral health and its association with systemic diseases, as well as determine any differences in knowledge due to gender, age, and educational level. Furthermore, this study will clearly address a gap in the existing literature by providing a clear rationale for examining this specific population, thereby enhancing understanding in this under-researched area.

## Materials and methods

This cross-sectional study was designed to assess public knowledge and awareness about oral health and its association with systemic diseases and was approved by the Biomedical Research Ethics Committee (approval no. HAPO-02-K-012-2023-02-1465).

A structured close-ended "true or false" and multiple choice questionnaire was developed in Arabic and consisted of two parts as follows: the first part included sociodemographic data about age, sex, occupation, and educational level, whereas the second part was designed to measure knowledge and awareness about oral health using 10 close-ended "true or false" and multiple choice questions (appendix). The questionnaire was designed following a careful review of the relevant literature and was validated using content validity by an expert in the field [9-23]. Furthermore, the questionnaire was piloted on 10% of the subjects - excluded from the study - and was amended based on their feedback. Cronbach’s alpha was used to assess the reliability and internal consistency of the questionnaire, which indicated good reliability and internal consistency (Cronbach’s alpha coefficient=0.752). An electronic version of the questionnaire was developed using Google Forms for distribution through different social media outlets, e.g., Twitter, and among patients visiting Umm AlQura University Dental Teaching Hospital, Makkah, Saudi Arabia from September 2023 to April 2024.

The sample size was calculated using the Epitools Epidemiological Calculator (Canberra, Australia: Ausvet) with a confidence level of 95%, 80% power, and a margin of error of 5%. This tool enabled the authors to determine that a sample size of 370 participants was sufficient to assess the knowledge of the Saudi population regarding oral health and its association with systemic diseases [19]. In this study, we excluded dental practitioners such as dentists, specialists, and dental students. We included all individuals who are 18 years and older. The study sample consisted of 370 subjects, inclusion criteria were adults aged 18 years or older, while dental practitioners such as dentists, specialists, and dental students were excluded from the study. Subjects answered a self-administered questionnaire of Knowledge, Attitudes, and Practices (KAP) about oral health and its association with systemic diseases.

To calculate the level of knowledge of the participants, a dichotomous scale was used and scoring was done by assigning points 1 for "true," 0 for "false. Furthermore, one score was given for each correct answer, and the total was calculated out of 10. For the purpose of statistical analysis, participants were considered to have good oral health knowledge and awareness if they answered 60% of the questions correctly.

The data were entered and coded in Excel (Redmond, WA: Microsoft Corp.), then analyzed using SPSS, version 29 (Armonk, NY: IBM Corp.). Descriptive statistics, chi-square, Fisher’s exact, Mann-Whitney U, and Kruskal-Wallis tests were used to analyze the data. The significance level was set at p<0.05.

## Results

A total of 322 subjects, agreed to participate in this study, most of which were female (214, 66.5%) and aged between 20 and 29 years (158, 49.1%) (Table 1). Further analysis showed that most participants were college graduates (239, 74.2%). Regarding their occupations, most participants were unemployed (194, 60.2%), while 76 (23.6%) worked in the government sector, 37 (11.5%) worked in the private sector (Table 1). Most participants (292, 90.7%) were healthy with only 11 participants (3.4%) reporting having asthma or other respiratory diseases (Table 1).

**Table 1:** Demographic characteristics of the participants and oral hygiene habits.

Demographic		Number	%
Age (years)	Below 20	54	16.8
	20-29	158	49.1
	30-39	55	17.1
	40-49	32	9.9
	50-59	20	6.2
	>60 (60-85)	3	0.9
Gender	Female	214	66.5
	Male	108	33.5
Educational level	Intermediate school	7	2.2
	Secondary school	47	14.6
	College	239	74.2
	Postgraduate studies (Master's and PhD)	22	6.8
	Others	7	2.2
Occupation	Government sector	76	23.6
	Private sector	37	11.5
	Private job	5	1.6
	Unemployed	194	60.2
	Retired	10	3.1
Do you clean your teeth using a brush and toothpaste?	Once a day	82	25.5
	Twice daily	145	45.0
	More than twice daily	57	17.7
	Irregular	33	10.2
	Use the Miswak only	5	1.6
Do you have any chronic diseases?	Nothing	292	90.7
	Diabetes	6	1.9
	Heart disease	1	0.3
	Hypertension	6	1.9
	Asthma and respiratory diseases	11	3.4
	Epidemic liver disease	2	0.6

Oral hygiene practices

Regarding regular oral hygiene practices, most participants (145; 45%) reported brushing their teeth twice a day, while 85 (25.5%) brushed their teeth once a day (Table 1).

Knowledge regarding the association between oral health and overall health

**Figure 1:**
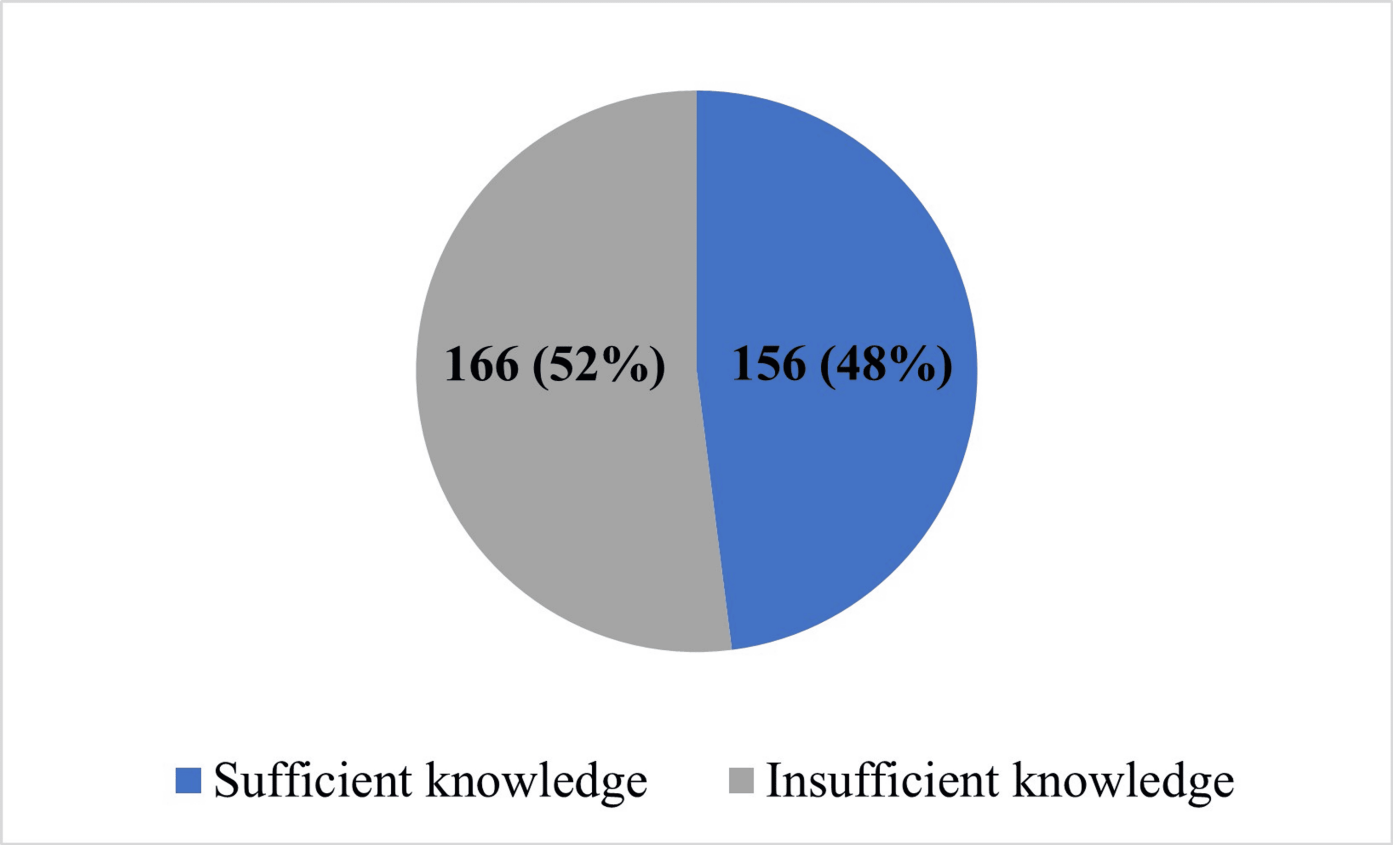
Level of knowledge regarding the impact of oral health on overall well-being among all participants. More than half of the participants showed insufficient knowledge regarding the impact of oral health on overall well-being.

The relationship between knowledge of oral health and general health was measured as shown in Table 2. The participants were classified as having "sufficient knowledge" or "insufficient knowledge" based on their responses to a set of questions. To be classified as having sufficient knowledge, participants had to answer at least six questions correctly, while those who answered fewer questions correctly were classified as having insufficient knowledge. More than half of the participants (166, 52%) were classified as having insufficient knowledge with median=5 and interquartile range (IQR)=4-8 (Figure 1).

**Table 2:** Participants’ knowledge of oral health and its association with systemic diseases.

Item		Number	%
1. Is a family member chronically ill?	Yes	150	46.6
	No	172	53.4
2. You should fully disclose your health to the dentist	True	283	87.9
	Wrong	6	1.9
	I don't know	33	10.2
3. Is there a close association between chronic diseases and oral health?	True	211	65.5
	Wrong	16	5
	I don't know	95	29.5
4. There is an association between heart disease and oral health	True	127	39.4
	Wrong	29	9
	I don't know	166	51.6
5. There is an association between diabetes and oral health	True	186	57.8
	Wrong	26	8.1
	I don't know	110	34.2
6. Is there an association between asthma and oral health?	True	106	32.9
	Wrong	50	15.5
	I don't know	166	51.6
7. There is an association between liver disease and oral health	True	143	44.4
	Wrong	33	10.2
	I don't know	146	45.3
8. There is an association between pregnancy health and oral health	True	191	59.3
	Wrong	23	7.1
	I don't know	108	33.5
9. It is important to maintain oral health	True	305	94.7
	Wrong	3	0.9
	I don't know	14	4.3
10. If most of your previous answers are correct, where did you get the information from?	The dentist	70	21.7
	The general practitioner	16	5
	Social media accounts	159	49.4
	The media	77	23.9

The associations between demographic data and level of knowledge

**Figure 2:**
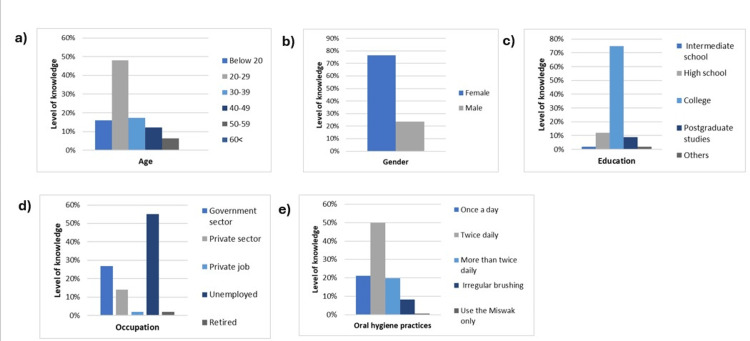
Level of knowledge regarding the impact of oral health on overall well-being among participants in correlation to different demographic data. The images show a comparison of the effects of different demographic variables on the level of knowledge regarding the impact of oral health on overall well-being - (a) age, (b) gender, (c) education, (d) occupation, and (e) oral hygiene practices.

The analysis revealed that there was no significant association between age and knowledge levels (p=0.537). Interestingly, the 20-29 years age group had the highest percentage of sufficient knowledge (48.1%), while the 50-59 years age group had the lowest percentage of sufficient knowledge (6.8%). There was also a statistically significant association between gender and knowledge levels (p<0.001). Specifically, females demonstrated a greater level of knowledge (76.3%) than males (23.7%) (Figure 2, panels a and b, and Table 3).

**Table 3:** The association between level of knowledge and demographic characteristics. *Fisher-Freeman-Halton exact test value. **Pearson chi-square test value. ***P≤0.05 indicates a significant difference.

Demographic factor		Level of knowledge		Test value	p-Value
		Insufficient	Sufficient		
Age (years)	Below 20	29 (17.5)	25 (16)	4.17*	0.537
	20-29	83 (50)	75 (48.1)		
	30-39	28 (16.9)	27 (17.3)		
	40-49	13 (7.8)	19 (12.2)		
	50-59	10 (6)	10 (6.4)		
	60<	3 (1.8)	0 (0)		
Gender	Female	95 (57.2)	119 (76.3)	13.1**	0.001***
	Male	71 (42.8)	37 (23.7)		
Education level	Intermediate school	4 (2.4)	3 (1.9)	3.5*	0.486
	High school	28 (16.9)	19 (12.2)		
	College	122 (73.5)	117 (75)		
	Postgraduate studies (Master's and PhD)	8 (4.8)	14 (9)		
	Others	4 (2.4)	3 (1.9)		
Occupation	Government sector	34 (20.5)	42 (26.9)	6.13*	0.186
	Private sector	15 (9)	22 (14.1)		
	Private job	2 (1.2)	3 (1.9)		
	Unemployed	108 (65.1)	86 (55.1)		
	Retired	7 (4.2)	3 (1.9)		
Do you clean your teeth using a brush and toothpaste?	Once a day	49 (29.5)	33 (21.2)	7.19*	0.120
	Twice daily	67 (40.4)	78 (50)		
	More than twice daily	26 (15.7)	31 (19.9)		
	Irregular	20 (12)	13 (8.3)		
	Use the Miswak only	4 (2.4)	1 (0.6)		
Do you have any chronic diseases?	No	151 (91)	141 (90.4)	0.03**	0.858
	Yes	15 (9)	15 (9.6)		
If most of your previous answers are correct, where did you get the information from?	The dentist	24 (14.5)	46 (29.5)	11.66**	0.009***
	The general practitioner	10 (6)	6 (3.8)		
	Social media accounts	92 (55.4)	67 (42.9)		
	The media	40 (24.1)	37 (23.7)		

The association between the participants' educational level and their knowledge was not significant (p=0.486); however, participants with a college degree exhibited a sufficient level of knowledge (75%). Conversely, the lowest percentage of sufficient knowledge was observed among individuals with intermediate school degrees and other degrees. Interestingly, participants with postgraduate degrees showed a lower level of knowledge than college graduates (Figure 2, panel c, and Table 3).

Further statistical analyses were conducted to investigate the potential association between occupation and knowledge level, indicating that there was no significant relationship between occupation and knowledge level (p=0.186). The data showed that 55.1% of the unemployed participants had sufficient knowledge levels, while 1.9% of the retired participants had lower levels of sufficient knowledge (Figure 2, panel d, and Table 3). Taken together, neither educational level nor occupation affects the participant’s knowledge; however, gender was associated with level of knowledge as females showed significantly more knowledge than males.

The association between participant’s oral hygiene practice and knowledge

Oral hygiene practices, namely, the frequency of brushing teeth with toothpaste, were investigated to establish their relationship with participants’ knowledge levels. There was no significant connection (p=0.120) with 50% of those with sufficient knowledge reporting brushing their teeth twice a day (Figure 2, panel e). These findings indicate that there is no significant relationship between oral hygiene behaviors and knowledge levels.

The association between chronic diseases and the knowledge level of participants

**Figure 3:**
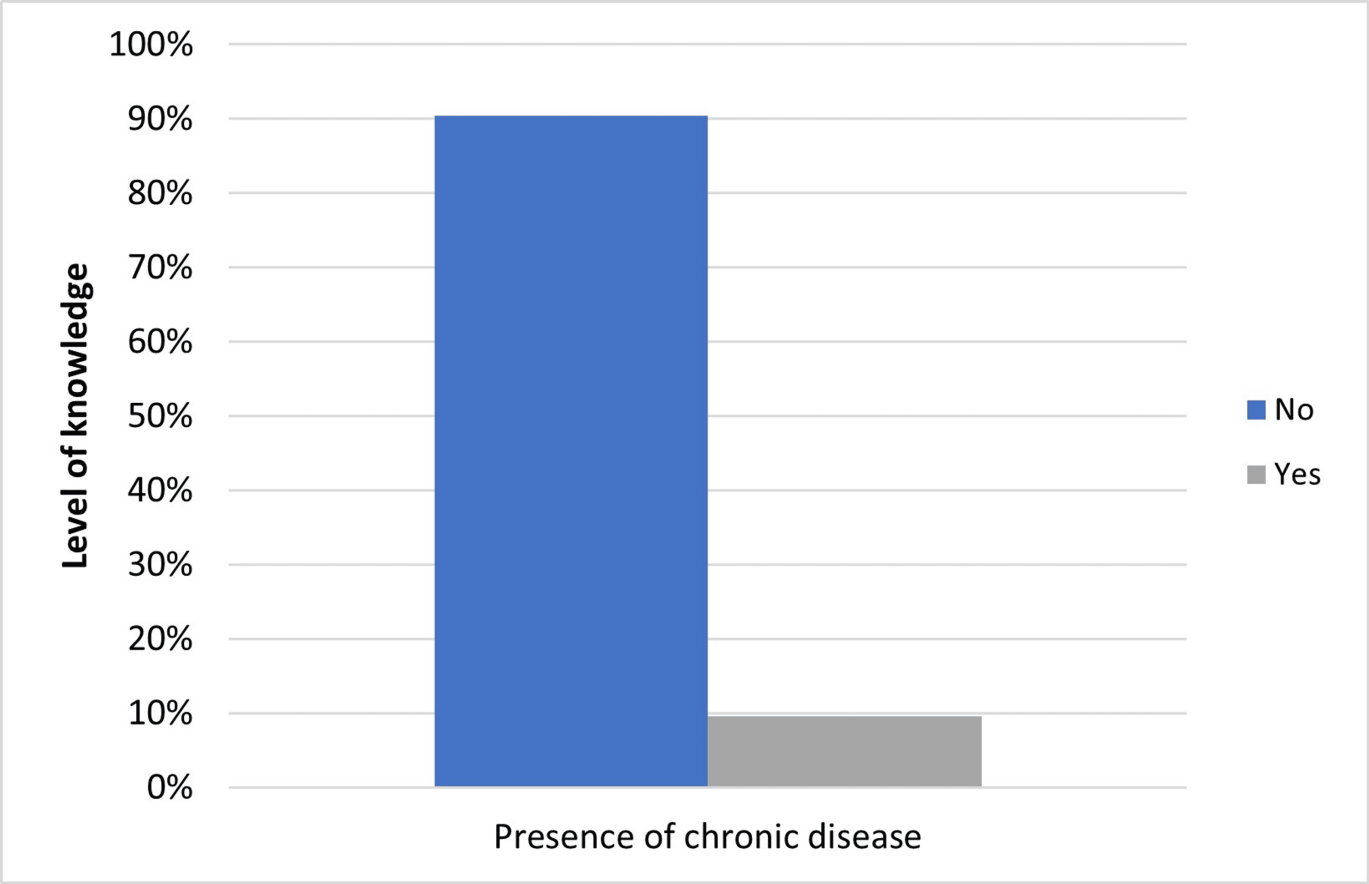
Association between level of knowledge regarding the impact of oral health on overall well-being and presence of chronic diseases. Presence of chronic diseases does not seem to affect the level of knowledge regarding the impact of oral health on overall well-being.

Since patients with chronic diseases visit healthcare providers more regularly, it is expected that they have a higher knowledge level than patients without chronic diseases. However, in this report, there was no significant correlation between the presence of chronic diseases and the level of oral health knowledge (p=0.858). The study revealed that individuals without chronic diseases had the highest percentage of sufficient knowledge (90.4%) when compared to individuals with chronic diseases (Figure 3 and Table 3). These results suggest that the presence of a chronic disease does not necessarily affect individuals’ levels of oral health knowledge.

The association between the source of information about oral health and the level of knowledge among participants

**Figure 4:**
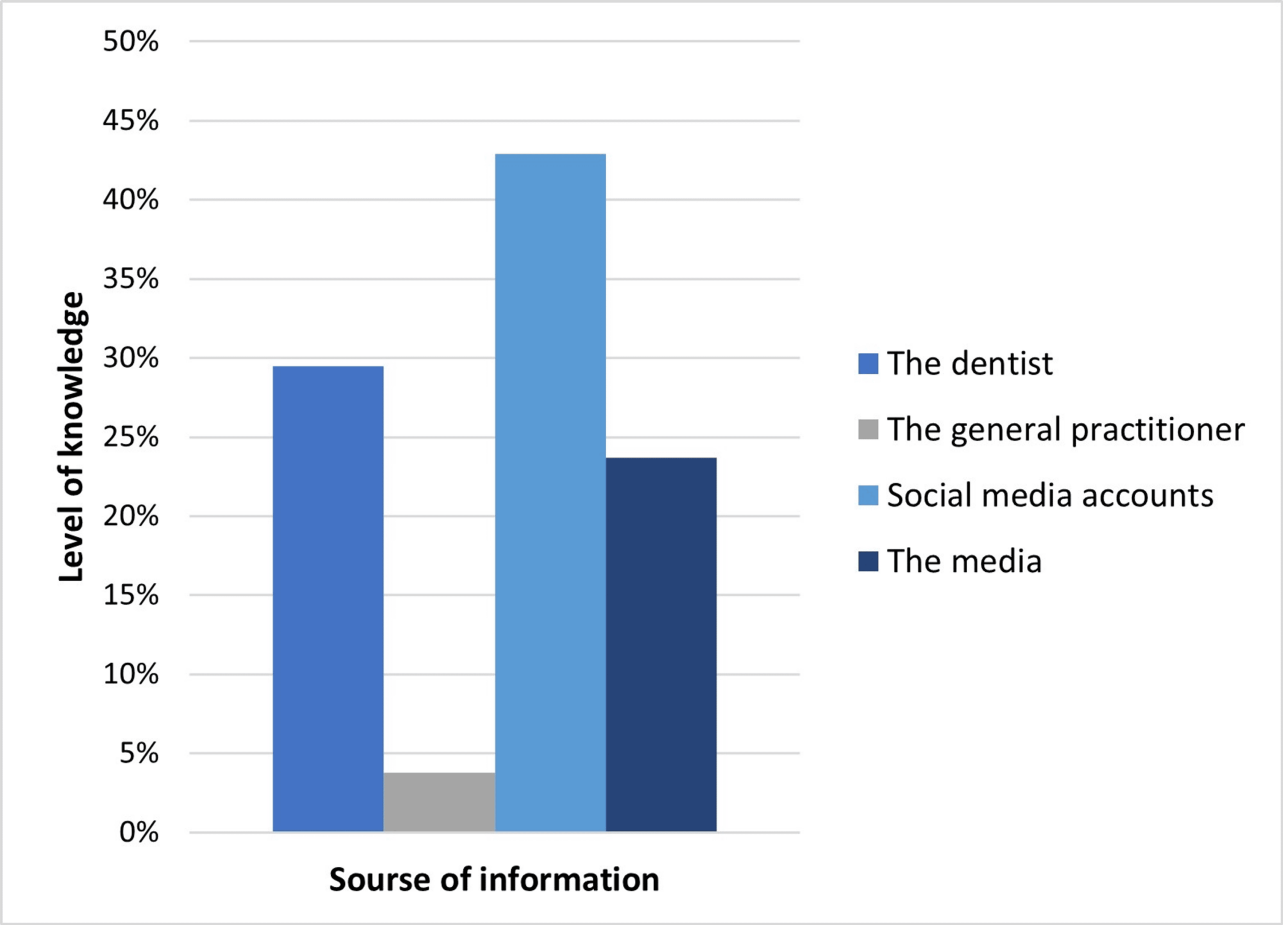
Level of knowledge regarding the impact of oral health on overall well-being according to source of information. Social media is the main source of knowledge regarding the impact of oral health on overall well-being among participants.

A significant association was found between the source of information about oral health and knowledge level (p=0.009), with most participants (42.9%) relying on social media for oral health information (Figure 4). It was observed that the participants who relied on social media had the highest level of sufficient knowledge, while only 3.8% of the participants who obtained their information from general practitioners had a lower level of sufficient knowledge (Table 3).

## Discussion

Oral health is a vital component of overall health and their relationship is complex. Numerous studies have identified a correlation between oral health and overall well-being. Moreover, research has established a link between dental health and the progression of chronic diseases. Chronic oral disorders can have a detrimental impact on general health and quality of life, while medically compromised individuals often exhibit poor dental health. For instance, there is an association between diabetes and various oral diseases, including periodontal disease, tooth loss, xerostomia, caries, burning mouth syndrome, taste and salivary gland dysfunction, and delayed wound healing [9,20,21]. Hence, the population must recognize the association between oral health issues and overall health, as enhanced awareness could lead to better preventive measures and prompt interventions, ultimately improving both oral and systemic health outcomes.

This study evaluated the correlation between different demographic data and the level of knowledge regarding the correlation between oral health and systemic health. In our cohort, female participants exhibited a greater level of knowledge regarding the correlation between oral health and systemic health, which was purportedly attributed to their inclination towards proactive health-seeking behavior and a heightened interest in healthcare compared to their male counterparts. These findings were consistent with previous results demonstrating that female participants exhibited a higher level of awareness compared to their male counterparts [22,23].

Regarding the relationship between educational attainment and levels of knowledge, individuals holding college degrees demonstrated higher levels of knowledge in comparison to those holding other types of degrees in line with a previous study [24]. Furthermore, participants with postgraduate degrees demonstrated lower levels of knowledge, possibly due to their area of study being distant from the field of oral health. Interestingly, these results do not align with previous studies that reported that participants with postgraduate degrees exhibit higher knowledge levels regarding oral health [25].

We also observed a notable discrepancy, where participants with diligent oral hygiene practices - specifically those who brushed their teeth twice daily - demonstrated inadequate knowledge regarding oral health. This finding contradicts a study conducted in the United States, where participants with better oral hygiene practices exhibited superior knowledge concerning oral health [26].

Individuals with chronic diseases had a lower level of knowledge compared to healthy participants. Similarly, studies conducted in Australia and Germany showed that participants with chronic diseases demonstrated inadequate knowledge regarding the correlation between cardiac complications, periodontal disease, and CVD [24,25]. Studies in India and Nigeria also revealed poor knowledge among participants regarding the oral manifestations of diabetes and the association between diabetes and periodontal disease [27,28].

The current study provided significant insights into the sources of oral health information among participants, with a notable majority relying on social media platforms. This highlights a shift in information consumption patterns, where digital channels increasingly influence health-related knowledge acquisition. However, it is concerning that only a minority sought information directly from healthcare providers, such as dentists or primary healthcare professionals.

The preference for social media as the primary source of oral health information raises important considerations regarding the quality, accuracy, and reliability of information accessed by individuals. Consistent with findings from previous studies, social media emerges as a dominant source of oral health information [29,30]. While these platforms offer convenience and widespread accessibility, they often lack the expert guidance and personalized advice that healthcare providers can offer. Information obtained from qualified professionals ensures accuracy, tailored recommendations, and personalized care plans crucial for maintaining optimal oral health.

Healthcare providers, particularly dentists, play a pivotal role not only in diagnosing and treating oral health conditions but also in educating patients about preventive measures and promoting oral hygiene practices. Other studies have found that participants rely on physicians as their primary source of health information [31,32]. Direct interaction with healthcare providers allows for comprehensive assessments, timely interventions, and the dissemination of evidence-based information tailored to individual needs. This personalized approach enhances health literacy and empowers individuals to make informed decisions about their oral health.

Recommendations

Based on survey findings, it is recommended to increase public awareness of the link between oral and systemic health through targeted educational programs, especially using popular social media platforms, as it was a primary source of information for participants. Furthermore, healthcare providers should be encouraged to discuss oral health during routine check-ups, particularly for patients with chronic conditions, to bridge the knowledge gap observed in the study.

Limitation

More than half of the study participants indicated that they did not have enough knowledge about oral health. However, the study did not assess the effectiveness of specific educational interventions. Future research would help bridge this gap in understanding how to improve awareness about oral health across different groups of people in Saudi Arabia. Another limitation of the study is the relatively small sample size, collected from only one location. Additionally, the reliance on self-reported data through questionnaires introduces the potential for response bias. To enhance the robustness and generalizability of the findings, it is crucial to expand the sample size and gather data from various locations or cities.

## Conclusions

Considering the limitations, this study's findings indicate a significant lack of awareness concerning oral health, especially among individuals with chronic conditions. This highlights the necessity for comprehensive health education and a deeper understanding of the connections between oral and systemic health. Furthermore, while social media is a prominent source of information, ongoing concerns about its accuracy and reliability remain. By addressing these issues through targeted educational initiatives and ensuring access to credible health information, individuals in Saudi Arabia can make informed decisions regarding their oral health, ultimately improving their overall well-being.

## Appendices

General knowledge and awareness of oral health in Saudi Arabia

We are a research team conducting a study to measure the knowledge of the Saudi community regarding the importance of oral health and its connection to overall health. Based on the results of this survey, we will provide recommendations and educational materials for all segments of society to raise awareness about the importance of oral and dental health.

Indicated questions:

1. Age:

(a) 10-19 years

(b) 20-29 years

(c) 30-39 years

(d) 40-49 years

(e) 50-59 years

(f) Over 60 years

2. Gender:

(a) Male

(b) Female

3. Educational level: 

(a) Intermediate

(b) High School

(c) University

(d) Postgraduate (Master’s-Ph.D.)

(e) Other

4. Occupation:

(a) Government sector

(b) Private sector

(c) Self-employed

(d) Unemployed

(e) Retired

5. Do you brush your teeth with toothpaste?

(a) Once daily

(b) Twice daily

(c) More than twice daily

(d) Irregularly

(e) Use only Miswak (natural toothbrush)

6. Do you have any chronic diseases? (Check all that apply)

(a) None

(b) Diabetes

(c) Heart disease

(d) High blood pressure

(e) Asthma and respiratory diseases

(f) Hepatitis and liver diseases

7. Does anyone in your family have chronic diseases?

(a) Yes

(b) No

8. True or false: you must fully disclose your health condition to the dentist.

(a) True

(b) False

(c) I don't know

9. True or false: there is a close link between chronic diseases and oral health.

(a) True

(b) False

(c) I don't know

10. True or false: there is a connection between heart disease and oral health.

(a) True

(b) False

(c) I don't know

11. True or false: there is a connection between diabetes and oral health.

(a) True

(b) False

(c) I don't know

12. True or false: there is a connection between asthma and oral health.

(a) True

(b) False

(c) I don't know

13. True or false: there is a connection between liver diseases and oral health.

(a) True

(b) False

(c) I don't know

14. True or false: there is a connection between pregnancy and oral health.

(a) True

(b) False

(c) I don't know

15. True or false: it is important to maintain oral health.

(a) True

(b) False

(c) I don't know

16. If most of your previous answers were "true," where did you obtain this information?

(a) Dentist

(b) General physician

(c) Social media

(d) Media

17. Which of the following health conditions do you think the dentist does not need to know about before treatment? (Select all that apply)

(a) Pregnancy and lactation

(b) Heart disease

(c) Asthma and respiratory diseases

(d) Diabetes

(e) Liver disease

(f) None of the above

18. Can the treatment plan for oral and dental health change due to your health condition?

(a) In all cases

(b) Often

(c) Rarely

(d) Will not change

(e) Only in emergency cases, not chronic diseases

## References

[1] Alazawi W, Bernabe E, Tai D (2017). Periodontitis is associated with significant hepatic fibrosis in patients with non-alcoholic fatty liver disease. PLoS One.

[2] Akinkugbe AA, Slade GD, Barritt AS (2017). Periodontitis and non-alcoholic fatty liver disease, a population-based cohort investigation in the Study of Health in Pomerania. J Clin Periodontol.

[3] Åberg F (2022). Oral health and liver disease: bidirectional associations - a narrative review. Dent J (Basel).

[4] Olander AE, Helenius-Hietala J, Nordin A, Savikko J, Ruokonen H, Åberg F (2023). Association between pre-transplant oral health and post-liver transplant complications. Transpl Int.

[5] Bozejac BV, Stojšin I, Ðuric M (2017). Impact of inhalation therapy on the incidence of carious lesions in patients with asthma and COPD. J Appl Oral Sci.

[6] Sim KY, Jang YS, Yoon NY, Park EC (2023). Association between Asthma and Oral Health Symptoms in Adolescents. Int J Environ Res Public Health.

[7] Ellepola AN, Samaranayake LP (2001). Inhalational and topical steroids, and oral candidosis: a mini review. Oral Dis.

[8] Nazir MA, AlGhamdi L, AlKadi M, AlBeajan N, AlRashoudi L, AlHussan M (2018). The burden of diabetes, its oral complications and their prevention and management. Open Access Maced J Med Sci.

[9] Lamster IB, Lalla E, Borgnakke WS, Taylor GW (2008). The relationship between oral health and diabetes mellitus. J Am Dent Assoc.

[10] Sohn HA, Rowe DJ (2015). Oral health knowledge, attitudes and behaviors of parents of children with diabetes compared to those of parents of children without diabetes. J Dent Hyg.

[11] Poudel P, Griffiths R, Wong VW, Arora A, Flack JR, Khoo CL, George A (2018). Oral health knowledge, attitudes and care practices of people with diabetes: a systematic review. BMC Public Health.

[12] Deghatipour M, Ghorbani Z, Ghanbari S, Arshi S, Ehdayivand F, Namdari M, Pakkhesal M (2019). Oral health status in relation to socioeconomic and behavioral factors among pregnant women: a community-based cross-sectional study. BMC Oral Health.

[13] Deghatipour M, Ghorbani Z, Mokhlesi AH, Ghanbari S, Namdari M (2022). Effect of oral health promotion interventions on pregnant women dental caries: a field trial. BMC Oral Health.

[14] Penmetsa GS, Meghana K, Bhavana P, Venkatalakshmi M, Bypalli V, Lakshmi B (2018). Awareness, attitude and knowledge regarding oral health among pregnant women: a comparative study. Niger Med J.

[15] Boggess KA, Urlaub DM, Moos MK, Polinkovsky M, El-Khorazaty J, Lorenz C (2011). Knowledge and beliefs regarding oral health among pregnant women. J Am Dent Assoc.

[16] Ruokonen H, Nylund K, Furuholm J (2017). Oral health and mortality in patients with chronic kidney disease. J Periodontol.

[17] Sanchez P, Everett B, Salamonson Y (2019). The oral health status, behaviours and knowledge of patients with cardiovascular disease in Sydney Australia: a cross-sectional survey. BMC Oral Health.

[18] Fardal Ø, Lygre H (2015). Management of periodontal disease in patients using calcium channel blockers - gingival overgrowth, prescribed medications, treatment responses and added treatment costs. J Clin Periodontol.

[19] (2024). Epitools - epidemiological calculators. https://epitools.ausvet.com.au/..

[20] de Medeiros TC, Areas E Souza A, Prates RC, Chapple I, Steffens JP (2022). Association between tooth loss, chronic conditions, and common risk factors: results from the 2019 Brazilian Health Survey. J Periodontol.

[21] Herrera D, Sanz M, Shapira L (2023). Association between periodontal diseases and cardiovascular diseases, diabetes and respiratory diseases: consensus report of the Joint Workshop by the European Federation of Periodontology (EFP) and the European arm of the World Organization of Family Doctors (WONCA Europe). J Clin Periodontol.

[22] Akl S, Ranatunga M, Long S, Jennings E, Nimmo A (2021). A systematic review investigating patient knowledge and awareness on the association between oral health and their systemic condition. BMC Public Health.

[23] Alessa N, Fathi W (2023). Assessment of dental patients’ awareness of the correlation between systemic and periodontal diseases: a questionnaire-based study. Cureus.

[24] Márquez-Arrico CF, Almerich-Silla JM, Montiel-Company JM (2019). Oral health knowledge in relation to educational level in an adult population in Spain. J Clin Exp Dent.

[25] Taheri A, Langarizadeh M, Dehkordi JG, Yousefianzadeh O (2020). Development of health literacy among postgraduate students: from information literacy perspective. J Educ Health Promot.

[26] Yuen HK, Wolf BJ, Bandyopadhyay D, Magruder KM, Salinas CF, London SD (2009). Oral health knowledge and behavior among adults with diabetes. Diabetes Res Clin Pract.

[27] Hollatz S, Wacker-Gussmann A, Wilberg S (2019). Awareness of oral health in adults with congenital heart disease. Cardiovasc Diagn Ther.

[28] Parakh MK, Kasi A, Ayyappan V, Subramani P (2020). Knowledge and awareness of oral manifestations of diabetes mellitus and oral health assessment among diabetes mellitus patients-a cross-sectional study. Curr Diabetes Rev.

[29] Afolabi O, Okeoghene OA (2017). Awareness and oral self-care practices of diabetic patients at a tertiary hospital in Lagos, Nigeria. Ann Med Health Sci Res.

[30] Amassi BY, Al Dakheel RS (2017). Oral hygiene practice of adult diabetic patients and their awareness about oral health problems related to diabetes. J Dent Oral Hyg.

[31] Pribble JM, Goldstein KM, Fowler EF, Greenberg MJ, Noel SK, Howell JD (2006). Medical news for the public to use? What’s on local TV news?. Am J Manag Care.

[32] Seematter-Bagnoud L, Santos-Eggimann B (2007). Sources and level of information about health issues and preventive services among young-old persons in Switzerland. Int J Public Health.

